# MR Defecography Improves Diagnosis of Postoperative Pelvic Floor Dysfunction After Gynecological Surgery

**DOI:** 10.3390/diagnostics15131625

**Published:** 2025-06-26

**Authors:** Rosa Alba Pugliesi, Marika Triscari Barberi, Giovanni Roccella, Giuseppe Gullo, Valentina Billone, Elena Chitoran, Gaspare Cucinella, Federica Vernuccio, Roberto Cannella, Giuseppe Lo Re

**Affiliations:** 1 Section of Radiology, Department of Biomedicine, Neuroscience and Advanced Diagnostics (BiND), University of Palermo, Via del Vespro 129, 90127 Palermo, Italy; rosaalba.pugliesi@unipa.it (R.A.P.); marikatriscari00@gmail.com (M.T.B.); giovannirocc@yahoo.it (G.R.); federica.vernuccio@unipa.it (F.V.); giuseppe.lore01@unipa.it (G.L.R.); 2 Department of Obstetrics and Gynecology, Villa Sofia Cervello Hospital, University of Palermo, 90146 Palermo, Italy; gullogiuseppe@libero.it (G.G.); valentina.billone@gmail.com (V.B.); gasparecucinella1@gmail.com (G.C.); 3 Department 10- General Surgery, “Carol Davila” University of Medicine and Pharmacy, 050474 Bucharest, Romania; 4 General Surgery and Surgical Oncology Department I, Bucharest Institute of Oncology “Al. Trestioreanu”, 022328 Bucharest, Romania

**Keywords:** pelvic floor dysfunction, MRI defecography, gynecological surgery, postoperative complications, pelvic organ prolapse, imaging techniques

## Abstract

Pelvic floor dysfunction (PFD) is one of the most significant postoperative consequences in gynecological surgery, leading to impaired bowel function, structural alteration, and reduced quality of life. The conventional technique using fluoroscopic defecography and perineal ultrasonography provides an incomplete assessment of multi-compartment defects and post-surgical changes. Magnetic resonance defecography (MRD) represents a valuable alternative imaging method in the assessment of PFD following gynecological surgery, increasing diagnostic accuracy and enabling personalized treatment planning. MRD achieves high-resolution multi-compartmental assessment of the pelvic floor in dynamic states. Particularly, it is able to detect postoperative complications such as mesh retraction, organ prolapse, and fistula formation, not visible to other modalities. This narrative review discusses the role of MRD in diagnosing PFD and its advantages in detecting functional and anatomical changes following gynecological surgery. This review also examined the ability of MRD to demonstrate surgical changes and its contribution to possible standardization in clinical practice.

## 1. Introduction

Female pelvic floor dysfunction (PFD) is a broad term encompassing various clinical conditions, most commonly including stress urinary incontinence (SUI), pelvic organ prolapse (POP), and anal incontinence [1]. Several risk factors contribute to PFD, including female gender, aging, menopause, obesity, pregnancy, and previous pelvic surgical procedures. All of them can compromise muscle tone, increase abdominal pressure, or damage the rectovaginal and pubovesical fasciae [2]. Genetic factors also play a role, as alterations in elastin and collagen contribute to urinary incontinence and POP [3,4].

PFD is particularly relevant following gynecological surgical intervention, especially those performed for POP [5]. While these surgical procedures are essential for addressing underlying conditions, they may inadvertently disrupt pelvic support structures, exacerbating or triggering new-onset PFD [6]. POP symptoms are frequently nonspecific, ranging from a sensation of vaginal heaviness to bladder and bowel dysfunction, further complicating clinical management [7,8].

Early and accurate diagnosis of PFD is critical, especially in postoperative patients, as delayed recognition may result in progressive symptom worsening and reduced quality of life [9]. Clinical evaluation, comprising physical, neurological, and digital rectal examinations, has inherent limitations, often underestimating prolapse severity and failing to detect complex issues like peritoneocele or evacuation disorders [10]. This highlights the growing importance of advanced imaging techniques in the diagnostic process.

PFD can be evaluated with different imaging modalities, each having distinct advantages. Urodynamic testing assesses bladder function, video-urodynamic and fluoroscopic studies provide evidence of prolapse and incontinence, cystoscopy is used for the diagnosis of bladder and urethral trauma, and ultrasonography (two- and three-dimensional) enables imaging of pelvic structures in real time [11].

**Figure 1:**
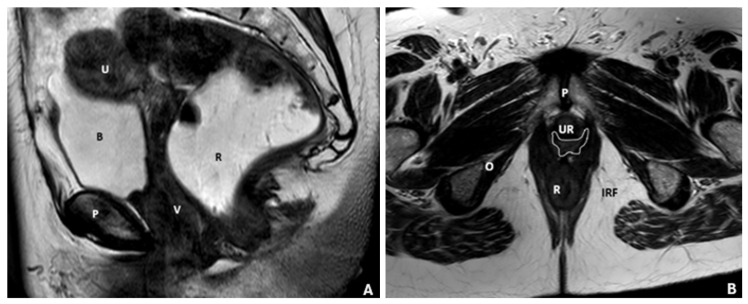
MRD of pelvic floor anatomy. (**A**) Sagittal T2-weighted turbo spin-echo (TSE) MRI illustrating normal female pelvic anatomy, clearly depicting the perineal body and levator plate. Anatomical landmarks include the bladder (B), uterus (U), vagina (V), rectum (R), and pubic bone (P). (**B**) Axial T2-weighted fast spin-echo (FSE) MRI demonstrating the pelvic floor anatomy. Identifiable structures include the pubic symphysis (P), rectus muscle (R), ischiorectal fossa (IRF), the normal butterfly-shaped vagina (outlined by a white line), urethra (UR), and the obturator muscle (O).

MR defecography (MRD) is an imaging technique with several advantages for the assessment of PFD (Figure 1), but its use is still limited. Unlike static MRI or fluoroscopic defecography, MRD provides dynamic, multi-compartmental images of the pelvic floor during varied functional states of rest, contraction, and defecation [12]. This feature facilitates better assessment of structural integrity and functional movement, and hence, detection of postoperative complications such as mesh retraction, erosion, fistula, and abnormal pelvic organ descent [13]. The higher soft tissue contrast provided by MRD is particularly beneficial in the assessment of surgical implants and the tissues around them, which provides essential information usually lost with other imaging modalities [14].

This narrative review discusses the role of MRD in diagnosing pelvic floor dysfunction, highlighting its advantages in detecting functional and anatomical changes after gynecological surgery, its ability to demonstrate surgical alterations, and its contribution to potential standardization in clinical practice.

## 2. Anatomical Structure of the Pelvic Floor

**Figure 2:**
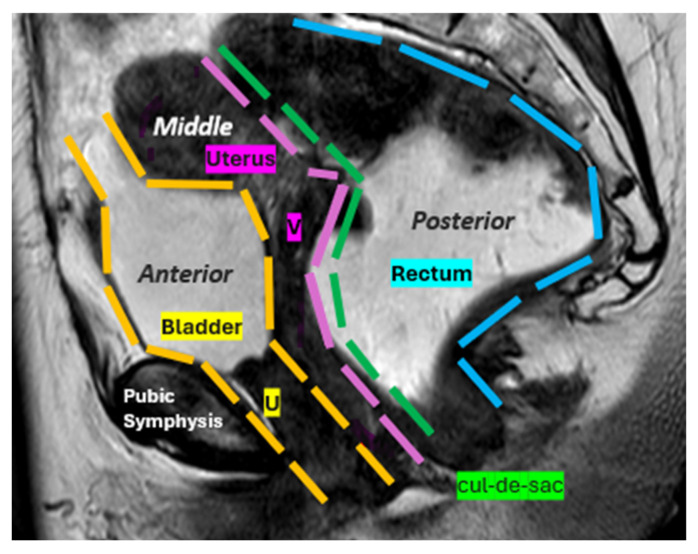
Female pelvic compartments. Sagittal T2-TSE image representing the three pelvic compartments: anterior, middle, and posterior. The anterior compartment, containing the bladder and urethra (U); the middle compartment, containing the uterus, cervix, and vagina (V); and the posterior compartment, containing the anus, anal canal, rectum (R), and sigmoid colon. A fourth “virtual” compartment called the cul-de-sac is also shown.

The pelvic floor is traditionally divided into three compartments in women (Figure 2), each encompassing distinct anatomical structures and supported by specific connective tissues and muscles [15]:

Anterior compartment: contains the urinary bladder and urethra, primarily supported by the pubocervical fascia, a component of the endopelvic fascia. Damage to this fascia can lead to urinary incontinence, cystocele, or urethral hypermobility.

Middle compartment: This includes the vagina, cervix, and uterus. The paracolpium and parametrium, as well as parts of the endopelvic fascia, provide support and play a crucial role in preventing genital organ prolapse.

Posterior compartment: comprises the rectum and anal canal, supported by the rectovaginal fascia and the perineal body, which prevents the widening of the urogenital hiatus. Structural damage in this region may result in rectocele or enterocele. A fourth virtual space, the cul-de-sac, may also be considered in imaging and clinical evaluation [16].

These compartments are maintained by three essential layers (Table 1):

Endopelvic fascia: the uppermost layer, encasing the pelvic diaphragm, acts as a scaffold for the pelvic organs, influencing their support and function [17].

**Figure 3:**
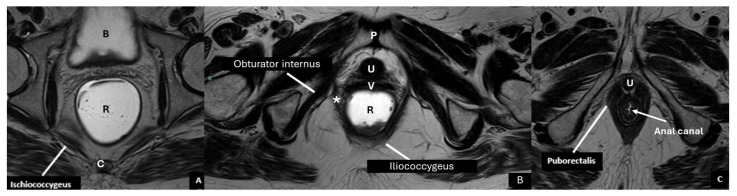
Pelvic muscles on axial MRI. Axial T2-weighted images (**A**–**C**) demonstrate normal pelvic diaphragm anatomy, including the iliococcygeus, ischiococcygeus, puborectalis (*), and obturator internus muscles. C = coccyx, P = pubic symphysis, R = rectum, U = urethra, B = bladder, V = vagina.

Pelvic diaphragm: consists of the levator ani muscles (puborectalis, pubococcygeus, and iliococcygeus) and the coccygeus muscle (Figure 3). The puborectalis forms a U-shaped sling around the rectum, playing a crucial role in maintaining the anorectal junction, while the iliococcygeus extends to the external anal sphincter, contributing to the levator plate that prevents posterior prolapse [18]. Additionally, the sacrospinous and sacrotuberous ligaments anchor the sacrum to the pelvis, increasing pelvic stability, while the cardinal ligament (Mackenrodt’s ligament) secures the uterus and upper vagina to the lateral pelvic walls, and the puboprostatic ligament in males connects the prostate to the pubic bone, supporting anterior pelvic structures [19].

**Figure 4:**
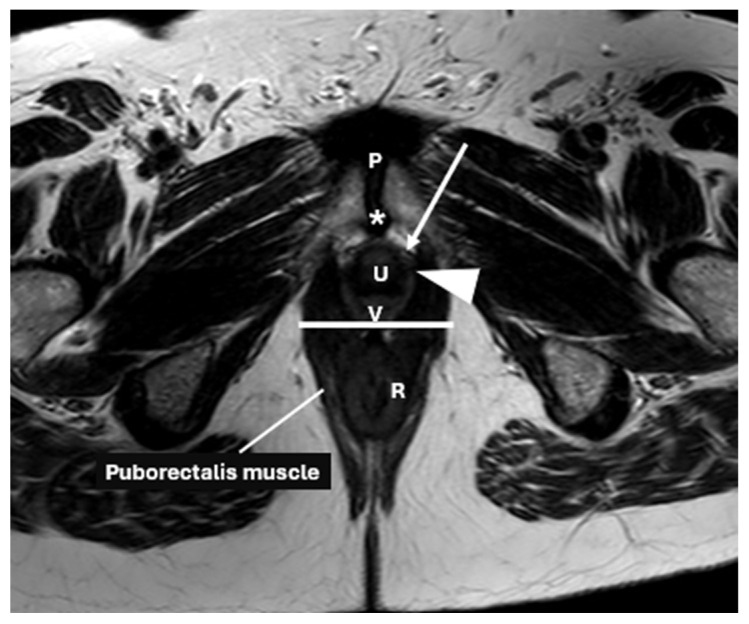
Normal female pelvic floor anatomy on axial MRI. Axial T2-weighted image shows the urogenital hiatus (white line) and key urethral support structures, including periurethral (arrow), paraurethral (arrowhead), and pubourethral (star) ligaments, with characteristic H-shaped vagina (V), urethra (U), pubic symphysis (P), and rectum (R).

Urogenital diaphragm: a triangular layer supporting the anterior pelvic organs which is present in the anterior and middle compartments, encircling the urethral sphincter and the deep perineal space [20]. The urogenital diaphragm forms the structure surrounding the urogenital hiatus, an opening in the pelvic floor that allows passage of the urethra and vagina (Figure 4). The urogenital hiatus is formed by the levator ani muscles, mainly the pubococcygeus and puborectalis muscles.

## 3. Importance of Early Diagnosis and Evaluation of PFD

PFD encompasses a range of disorders affecting the proper function of the pelvic floor muscles, ligaments, and connective tissues, often leading to symptoms such as SUI, POP, and defecatory dysfunction [21].

### 3.1. Role of Surgery

Gynecological surgical procedures play a crucial role in the management of both benign and malignant conditions of the female reproductive system [22]. Fertility-sparing surgery is an important option for young women with early-stage gynecological cancers, aiming to preserve reproductive potential while treating cancer [23].

The pelvic floor depends on a complex network of muscles, ligaments, and connective tissue for continence and support, which can be compromised by surgery like radical trachelectomy involving removal of the cervix and paracervical tissues around it, which may cause disruption of utero-sacral ligaments and deterioration in pelvic support [24,25]. Similarly, ovarian cancer operations, even those performed by fertility-sparing techniques, can jeopardize significant supportive structures and predispose patients to vault prolapse [26].

Long-term follow-up is essential for women undergoing fertility-sparing surgery, not only to monitor for cancer recurrence but also to address emerging pelvic floor issues. Standardized assessment tools, such as the Pelvic Floor Distress Inventory (PFDI) and the International Consultation on Incontinence Questionnaire (ICIQ), facilitate ongoing evaluation [27,28]. Early treatment by intervention, such as pessary or pelvic floor physical therapy, may halt the progression of symptoms and ensure long-term pelvic function [27]. In cases of severe POP, surgical intervention is often necessary. Colposacropexy is still the gold standard for repair of apical prolapse and is supplemented by minimally invasive surgery—laparoscopic and robotic—radically transforming treatment [29,30]. Laparoscopic colposacropexy is defined by reduced blood loss, reduced hospital stays, and faster recovery compared to open surgery [31,32], while robotic colposacropexy offers more precision but is often described by longer operating times and higher postoperative pain scores [14,33].

### 3.2. Imaging Techniques for Diagnosing PFD

The accurate diagnosis and assessment of PFD rely on various imaging techniques, each offering unique advantages for evaluating pelvic floor anatomy and function [34], summarized in Table 2, which also categorizes the types of POP defects with corresponding diagnostic and treatment options.

The initial evaluation of urinary incontinence in women typically involves a comprehensive approach that includes clinical history, physical examination, urinalysis, and postvoid residual urine measurement [35]. While basic assessments may be sufficient for patients with stress incontinence linked to urethral hypermobility, more complex cases necessitate further diagnostic testing [36]. The International Scientific Committee of the Third International Consultation on Urinary Incontinence recommends urodynamic testing for patients considering interventional treatments [37,38]. This test provides a detailed understanding of bladder function, detecting abnormalities such as detrusor overactivity, impaired compliance, and voiding dysfunction.

In the context of POP, lower urinary tract symptoms often accompany anatomical changes [39]. Urodynamic findings may reveal obstruction caused by cystoceles or other forms of prolapse [40]. Flow rates and pressure/flow studies provide valuable insights into the degree of bladder outlet obstruction. Video-urodynamic and fluoroscopic studies further confirm incontinence and bladder hypermobility while characterizing the nature and severity of organ prolapse [41]. These imaging modalities guide preoperative planning by identifying both the primary defect and any concomitant functional abnormalities [42].

Cystoscopy, although controversial for routine evaluation, remains a useful tool in certain scenarios [43]. Intraoperative cystoscopy assists in identifying bladder or urethral perforations and assessing ureteric patency during pelvic surgical procedures, especially those involving high-grade prolapse or multiple prolapsing organs [44].

Ultrasonography offers a non-invasive, radiation-free imaging option for the bladder and urethra [45]. Transabdominal, transperineal, translabial, transvaginal, and transrectal approaches allow real-time assessment of bladder neck mobility, stress incontinence, bladder wall thickness, levator ani activity, and prolapse quantification [46].

Advancements in ultrasound technology have introduced three-dimensional (3D) imaging, which combines multiple two-dimensional (2D) planes to provide a more comprehensive view of pelvic structures [47]. 3D ultrasound enhances the visualization of the urethra, levator ani complex, paravaginal supports, and synthetic implants, improving diagnostic precision for complex PFD cases [48]. Despite its utility, ultrasound is generally reserved for patients with inconclusive findings from primary evaluations, serving as a supplementary rather than a first-line diagnostic tool [45].

Additional diagnostic techniques include electromyography, which assesses pelvic floor muscle innervation, and defecography, particularly MRD, useful in evaluating rectoceles and enterocele. These methods add value in cases of defecatory dysfunction or complex multicompartment prolapse.

### 3.3. Role of MRI in Diagnosing Postoperative Complications

MRI has emerged as an indispensable tool in the diagnosis and evaluation of postoperative complications following gynecological surgical interventions, particularly for assessing PFD [48]. Unlike other conventional imaging techniques, MRI offers high soft tissue contrast and multiplanar imaging, enabling comprehensive assessment of both anatomical and functional abnormalities [49,50]. This high-resolution visualization is especially critical in postoperative settings, where subtle complications, such as mesh-related issues, organ prolapse, and fistulas, may be challenging to detect with other exams [51]. Moreover, MRI does not involve ionizing radiation, making it particularly advantageous for younger patients or those requiring multiple follow-up evaluations [52].

**Figure 5:**
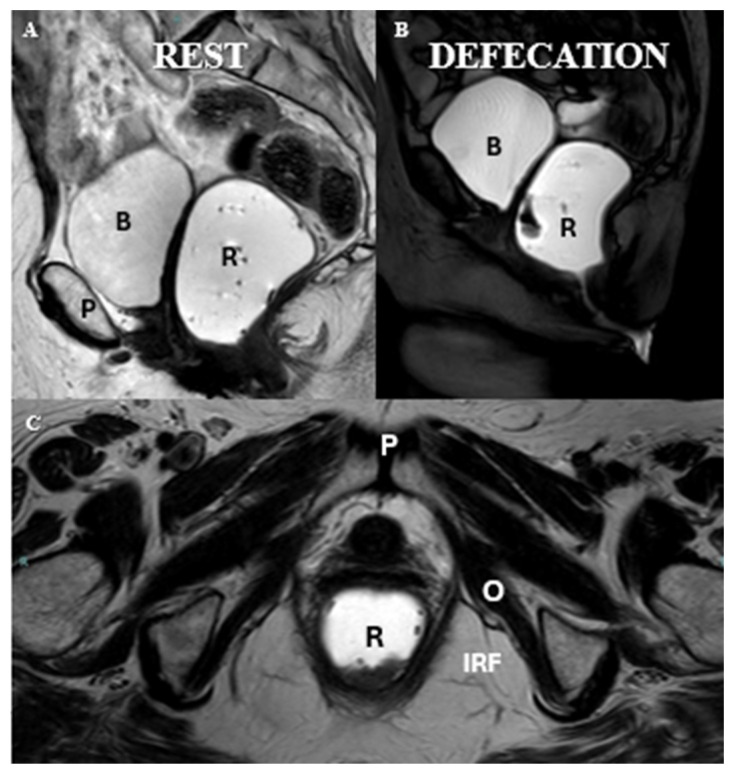
MR defecography in a post-hysterectomy patient. (**A**) Sagittal T2-weighted turbo spin-echo (TSE) MRI illustrating the female pelvic anatomy at rest; (**B**) sagittal balanced turbo field echo (BTFE) sequence during the evacuation phase; (**C**) axial T2-weighted turbo spin-echo (TSE) sequence showing the pelvic floor anatomy. Identifiable structures include the pubic symphysis (P), rectum (R), ischiorectal fossa (IRF), bladder (B), and the obturator muscle (O).

MRD is the foundation of dynamic pelvic floor assessment among all MRI applications. MRD consists of a sequence of image acquisitions during rest, contraction (squeeze), and straining or defecation, allowing real-time visualization of pelvic organ mobility and interaction [13]. Dynamic evaluation is the key to identifying the functional dysfunction in the postoperative period and anatomical anomalies (Figure 5).

For instance, in patients with pelvic organ prolapse treated with colposacropexy, MRD can detect abnormal vaginal descent, even in the presence of mesh, indicating mesh failure or incomplete repair [53,54]. MRD is also able to identify newly developed dysfunctions such as rectocele or enterocele, which may result from surgical trauma or altered pelvic biomechanics.

MRD can simultaneously examine all three pelvic compartments: anterior (bladder and urethra), middle (vagina and uterus), and posterior (rectum and anal canal) to diagnose complex, multi-compartmental dysfunctions that are often associated with each other following gynecologic procedures [12]. One of the critical factors in evaluating the degree of severity of prolapse and management strategies is the degree of organ descent and anorectal angles, which can be objectively assessed with MRD [55]. This is facilitated by standardized anatomical reference lines, like the mid-pubic line (MPL) and the pubococcygeal line (PCL), which allow measurement of the descent of pelvic organs and characterization of functional abnormalities such as anismus or rectal intussusception [56]. These measurable indicators facilitate customized treatment strategies and improve diagnostic reproducibility.

In addition to functional evaluation, both static and dynamic MRI sequences allow for the identification of structural complications, including mesh erosions, retractions, fluid collections, abscesses, and fistulas [57,58]. With its ability to view inflammatory changes and tract formation with high-resolution imaging, MRD is especially useful in delineating complex postoperative complications. Above all, unlike conventional fluoroscopic defecography based on barium-based contrast media, which is not suitable for all patients, MRD does not include intraluminal contrast, but instead uses a gel to distend the lumen [59]. Moreover, sufficient resolution of soft tissue to differentiate from non-radiopaque material, such as synthetic meshes or pelvic floor muscles, is unavailable with conventional fluoroscopy.

It should be noted that MRD can be unsuitable for patients with MRI contraindications, in which case fluoroscopic defecography remains an essential alternative for functional evaluation [59]. Structured templates with objective prolapse measurements, functional observations, and postoperative data need to be developed to enhance diagnostic accuracy and communication [60]. Further challenges include the relatively high cost and limited availability of MRD, which may restrict its use in resource-limited settings. Overcoming such barriers with protocol optimization and cost-effectiveness studies will be critical for improving clinical accessibility [61]. Lastly, despite early data indicating that MRD may improve risk stratification and inform postoperative management, rigorous multicenter studies are needed to validate the prognostic capacity of MRD and its incorporation into routine disease management algorithms. Advancing predictive modeling and enveloping MRD within routine clinical workflow will necessitate collaboration between multiple disciplines, including radiologists, gynecologists, colorectal surgeons, and data scientists.

## 4. MR Imaging Protocol

### 4.1. Patient Preparation

Patient cooperation is crucial for the success of the examination. Adequate preparation and clear communication regarding the aims of the study enhance patient compliance. Although no extensive preparation is required, such as enemas, intravenous contrast administration, or fasting, the patient should be advised to void approximately two hours before the study.

### 4.2. Equipment and Positioning

It is recommended to use a 1.5 T (or higher) MR scanner equipped with a multicoil array positioned around the pelvis [62]. The patient should be placed in the supine position with knees elevated. While this positioning does not fully replicate the physiological evacuation posture, it offers good sensitivity and provides clinically relevant findings for pelvic floor analysis [63].

### 4.3. Contrast and Opacification

Rectal distension (120–250 cc of ultrasound gel) is necessary to adequately visualize the rectal wall and its dynamics. Vaginal filling with 20 cc of ultrasound gel may also be useful to strengthen the delineation of adjacent structures.

### 4.4. Imaging Sequences

**Figure 6:**
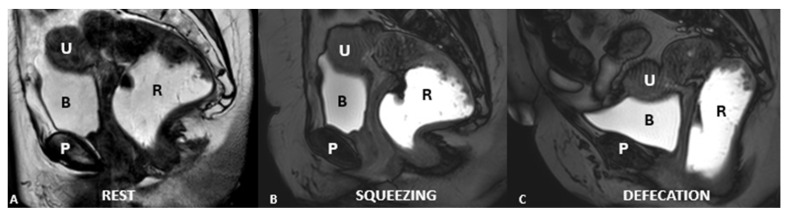
Normal position at rest (**A**), during straining (**B**), and defecation (**C**). There is a mild descent of all the three compartments (urinary bladder, vaginal vault, and anorectal junction). Identifiable structures include the pubic symphysis (P), rectum (R), bladder (B), and uterus (U).

The MRI protocol consists of both static and dynamic sequences (Table 3) and typically lasts approximately 30 min, depending on institutional protocols and patient compliance (Figure 6):

Static Imaging:
  - High-resolution T2-weighted images in three orthogonal planes: axial, sagittal, and coronal.
Dynamic Imaging:
  - Steady-state or balanced steady-state free precession (SSFP) sequences.
  - Acquired in the sagittal plane during various maneuvers: straining, squeezing, and evacuation.
  - Optional dynamic sequences in axial and coronal planes during straining may be considered to further evaluate complex pelvic floor dysfunctions.

### 4.5. Key Maneuvers

Valsalva maneuver (strain or bear down): Involves bearing down on the pelvic floor maximally with a closed anal sphincter, without evacuating any rectal contents [64]. This maneuver provides critical information regarding the degree of organ prolapse and the competence of the pelvic floor muscles.

Kegel maneuver (squeeze): Entails the maximal pelvic floor squeeze as if trying to prevent the passage of feces or urine [64]. This maneuver is instrumental in assessing the contraction strength of the puborectalis muscle and the ability to elevate the pelvic floor.

Defecation: Requires maximal bearing down with an open anal sphincter and complete evacuation of rectal contents. It is used to evaluate the function of the internal and external anal sphincters and the coordination of pelvic floor muscles during evacuation.

The anorectal angle (normal value in women is 108–127° and about 101° in men) becomes more acute during squeezing or Kegel muscle contraction, secondary to the shortening of the puborectalis muscle and the elevation of the anorectal junction [65]. Conversely, during defecation, the puborectalis muscles and levator plate typically relax, resulting in the expected widening of the anorectal angle [66]. The absence of puborectalis relaxation and anorectal widening during defecation may indicate defecatory dysfunction. In women, the anorectal angle decreases during contraction and increases during defecation by approximately 15–20° [67].

**Figure 7:**
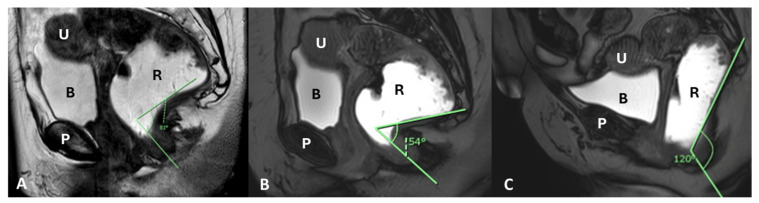
Sagittal T2-weighted MR defecography images demonstrate anorectal angle dynamics. (**A**) At rest post-rectal gel filling, the angle measures 81°, with the anorectal junction at the pubococcygeal line (PCL). (**B**) During pre-defecation contraction, the angle narrows to 54°. (**C**) During defecation straining, rectal prolapse widens the angle to 120°, with a 6 cm anorectal junction descent. Identifiable structures include the pubic symphysis (P), rectum (R), bladder (B), and uterus (U).

This stepwise assessment of both pre-defecation and defecation phases is essential in MRD, as it allows precise differentiation between muscular contraction disorders and mechanical pelvic organ prolapse, offering a comprehensive functional and anatomical diagnosis (Figure 7).

Key pelvic floor measurements, specifying the imaging planes and examination phases essential for evaluating dynamic and static pelvic floor dysfunctions, are summarized in Table 4.

### 4.6. Imaging Analysis of the Pelvic Floor

The MRD imaging process involves two key components: morphological and dynamic assessment.

Morphological assessment: focuses on muscle trophicity, particularly the iliococcygeus and puborectalis muscles, better visualized in coronal and axial views [68]. The puborectalis wraps posteriorly around the rectum, forming a muscular sling, while the iliococcygeus originates from the tendinous arch and extends to the anal sphincter [58].

**Figure 8:**
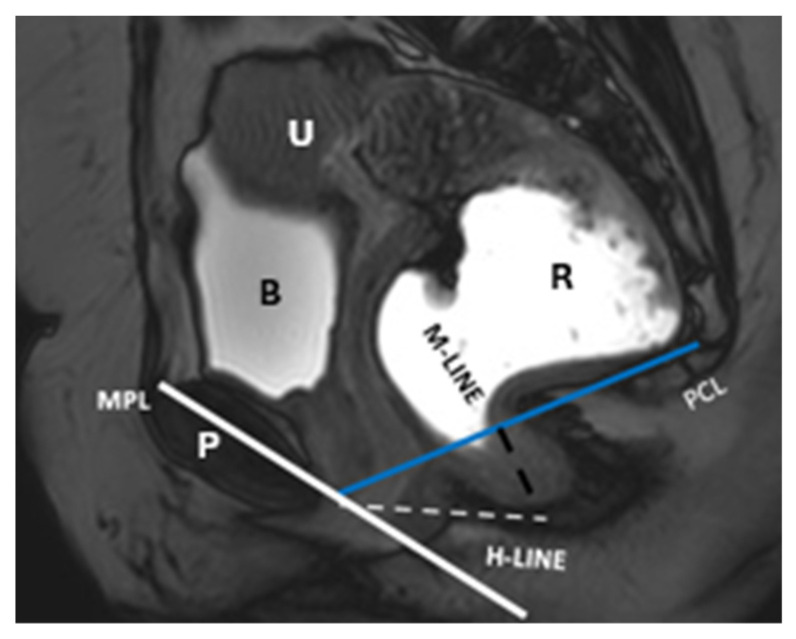
Sagittal T2-weighted MR defecography at squeeze shows normal pelvic anatomy. The pubococcyxgeal line (PCL), H line, M line, and mid-pubic line (MPL) are displayed. The anorectal junction aligns with the PCL, the H and M lines measure within normal limits, and no pelvic floor descent or organ prolapse is observed. Identifiable structures include the pubic symphysis (P), rectum (R), bladder (B), and uterus (U).

Dynamic assessment: evaluates pelvic organ descent and muscle contractions at rest, during straining, and evacuation [69], utilizing key anatomical reference lines and angles (Figure 8) (Table 4).

Pubococcygeal line (PCL)**:** Drawn from the inferior pubic border to the last junction between the first and second coccygeal segments, marking the plane of pelvic floor muscle attachment. Minimal organ descent (less than 1 cm below the PCL) is typical in healthy individuals [70,71].

H line (hiatal line): Measures the levator hiatus width from the inferior pubis to the anorectal junction, with a normal range of ≤5 cm [70,71].

M line (muscle line): Perpendicular to the PCL, measuring the descent of posterior pelvic organs. The normal range is ≤2 cm [70,71].

Mid-pubic line (MPL): The MPL serves as a reference line for assessing POP, similar to the PCL. It is defined on sagittal MRI as a line extending caudally through the axis of the mid-pubic symphysis. A 90° angle is measured between the MPL and the bladder, vaginal vault, and anterior anorectal junction to evaluate prolapse [70,71]. Additionally, it aligns with the midsagittal aspect of the pubic bone at the approximate level of the vaginal hymen.

Anorectal angle: Formed between the posterior distal rectum and the anal canal’s central axis. The normal angle ranges from 108° to 127°, decreasing by 15–20° during levator ani contraction [70,71].

## 5. The HMO System

To standardize MRI interpretation of pelvic floor dysfunction, the HMO (H line, M line, organ prolapse) system was developed [72]. This approach relies on a midsagittal rapid half-Fourier T2-weighted image taken during maximal strain. Three anatomical reference points are defined:

- Point A: The inferior margin of the symphysis pubis
- Point B: The convex posterior margin of the puborectalis muscle sling
- Point C: The junction between the first and second coccygeal segments

The PCL is drawn between points A and C, while the puborectal hiatus line (H line) extends from A to B, enabling the assessment of pelvic floor relaxation [72,73]. The degree of hiatal widening (anteroposterior dimension during straining) and hiatal descent are quantified, offering objective metrics for classifying pelvic floor dysfunction severity [62].

The organ prolapse (O) component of the HMO system measures the shortest distance between the most caudal part of an organ during the Valsalva maneuver and the H line [16], enabling standardized classification and grading of pelvic organ prolapse.

This structured approach enhances diagnostic accuracy and reproducibility across clinical settings.

## 6. Evaluating Pelvic Floor Disorders with MR Defecography

Pelvic floor functional disorders encompass a spectrum of conditions affecting the urethra, urinary bladder, vaginal vault, uterus, and rectum [23]. These disorders often result from complex interactions between anatomical structures and neuromuscular functions, necessitating a thorough understanding for accurate diagnosis and treatment [30]. Among these disorders, POP and pelvic floor relaxation are prominent, often coexisting in patients and contributing to varied symptomatology [12].

Diagnosis of PFD in the postoperative patient for gynecologic surgery remains a modern clinical challenge in comprehending the interrelated amalgamation of anatomic alteration, functional loss, and potential operation complications [1]. Traditional tests such as physical examinations, fluoroscopic defecography, and perineal ultrasonography provide an incomplete understanding of postoperative adaptation, as physical exams fail to detect subtle multi-compartment defects and fluoroscopic techniques, though useful for dynamic assessment, lack the soft tissue contrast needed to evaluate surgical changes, mesh placement, and complications [74,75]. MRD addresses these limitations by offering a high-resolution, multi-planar evaluation of pelvic anatomy and dynamic function (Table 5).

### 6.1. Anterior Compartment

The anterior compartment holds particular significance in urogynecology, as it involves crucial structures like the bladder and urethra [13,76]. Patients commonly present with SUI, a distressing symptom that requires precise clinical and radiological assessment.

#### Cystoceles

**Figure 9:**
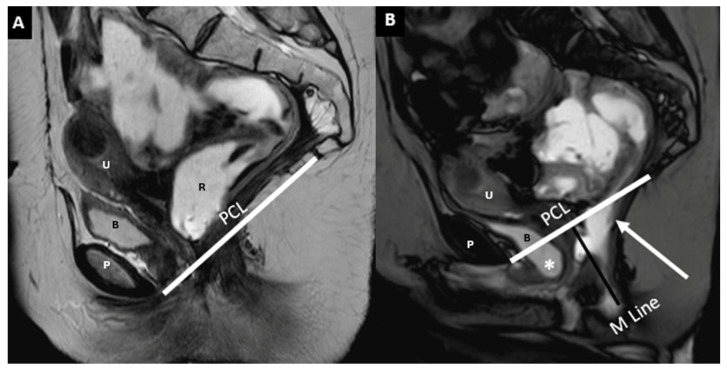
Sagittal midline images pre-evacuation (**A**) and during evacuation (**B**) illustrate descending perineal syndrome with tricompartimental prolapse—cystocele (star), uterine, and rectal prolapse—alongside anterior rectocele and rectal mucosa intussusception (white arrow). The evacuation phase is essential for revealing the full extent of pelvic floor dysfunction. M-line (dark) and PCL (white) are shown. Identifiable structures include the pubic symphysis (P), rectum (R), bladder (B), and uterus (U).

A cystocele is defined by the prolapse of the bladder through its respective hiatus in the anterior compartment. While mild cases are often diagnosed clinically, high-grade cystoceles necessitate MRD to identify associated urethral prolapse and bladder rotation. In severe cases, the posterior bladder wall descends more than the anterior wall, causing downward and clockwise bladder rotation along with urethral hypermobility [77]. This transverse orientation of the urethra may paradoxically mask SUI, only unmasked after bladder prolapse repair. High-grade cystourethroceles often involve multicompartment organ prolapse and pelvic floor relaxation, complicating the clinical picture. MRD aids in preoperative planning by assessing the degree of prolapse (Figure 9) and identifying complications like ureteral obstruction or hydronephrosis.

### 6.2. Middle Compartment

The middle compartment comprises the parametrium, paracolpium, rectovaginal fascia, and pubocervical fascia, which collectively support pelvic organs [13,76]. Dysfunction within this compartment can result in vaginal and uterine prolapse.

#### 6.2.1. Uterine Prolapse

Uterine prolapse, or procidentia, involves the descent of the uterus into the vaginal canal, sometimes progressing to complete eversion of the vaginal walls (Figure 9). MRD provides precise grading of uterine prolapse, guiding individualized treatment plans. Clinicians use these imaging findings to evaluate the extent of uterine descent and plan surgical or conservative interventions accordingly.

#### 6.2.2. Vaginal Prolapse

Vaginal prolapse, often linked to apical prolapse following hysterectomy, results from paracolpium injury. Diagnosis is complicated by coexisting conditions, such as large uterine fibroids. MRD helps assess the vaginal apex’s position, ensuring it remains at least 1 cm above the PCL in post-hysterectomy patients.

### 6.3. Posterior Compartment

**Figure 10:**
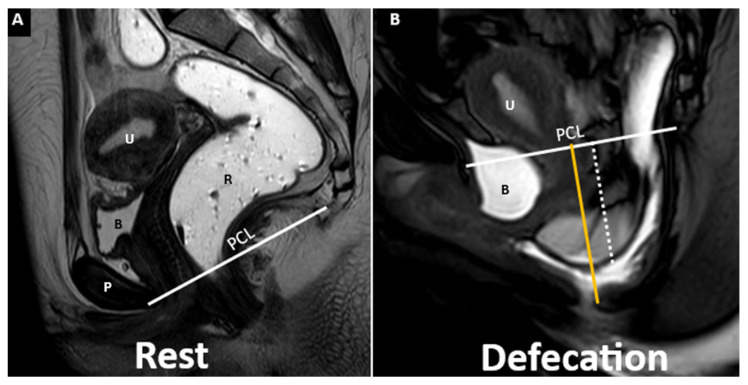
At rest (**A**), pelvic organs maintain normal positions. During defecation (**B**), notable descent of the peritoneal sac with small bowel loops defines an enterocele (white dotted). Associated findings include bladder and anorectal descent, anterior rectocele, rectorectal intussusception, cystocele, hysterocele, and severe elitrocele. M-line (orange) and PCL (white) are indicated. Identifiable structures include the pubic symphysis (P), rectum (R), bladder (B), and uterus (U).

Posterior compartment disorders are particularly relevant in women following gynecological surgical procedures, such as hysterectomy or POP repair, due to weakened pelvic floor structures [13,76]. Pelvic floor weakness after surgery may lead to the protrusion of the rectum, small bowel, or peritoneal contents through the vaginal or rectal lumen, resulting in rectocele, enterocele, sigmoidocele, or peritoneocele (Figure 10). Patients who have undergone hysterectomy are particularly prone to vaginal prolapse. Additionally, vaginal descent creates a retrovaginal space, predisposing them to the development of enterocele and peritoneocele [78].

Importantly, rectal prolapse frequently coexists with POP, with reported concomitance rates of between 21 and 34% in post-surgical patients [79,80]. In these cases, simultaneous prolapse of both the rectum and adjacent pelvic organs can occur, complicating symptoms and management.

Management strategies in post-surgical patients depend on symptom severity and prolapse grading. While mild cases may respond to conservative treatments, such as pelvic floor physiotherapy or biofeedback, surgical repair is often necessary in moderate to severe cases, particularly when multiple compartments are involved [31,69]. The increasing recognition of these combined defects has led to a growing trend of multidisciplinary approaches and combined surgical interventions. The frequency of combined POP and rectal prolapse repair surgeries increased from 2.6% to 7.0% over the past decade, particularly benefiting post-gynecological surgery patients with altered pelvic anatomy by reducing anesthesia risks, hospitalization, and recovery time while improving overall outcomes [78].

#### 6.3.1. Enterocele

Among posterior compartment disorders, enteroceles—commonly seen post-hysterectomy due to disruption of the rectovaginal and pubocervical fascia—often present with symptoms such as low back pain, dyspareunia, vaginal bulging, or bowel obstruction after defecation, and MRD aids in differentiating herniation types to guide peritoneal surgical repair [81,82].

#### 6.3.2. Sigmoidocele

Sigmoidocele, caused by rectosigmoid colon herniation into the posterior vaginal wall and causing symptoms like incomplete evacuation and pelvic discomfort. MRD can determine herniation severity and guide tailored treatments, including potential surgical resection of the rectosigmoid colon [70].

#### 6.3.3. Peritoneocele

Peritoneocele, the herniation of peritoneal contents into the rectovaginal fascia, typically presents with symptoms such as bowel obstruction or vaginal and pelvic pressure during evacuation. MRD enables precise anatomical and dynamic evaluation essential for surgical planning [70].

#### 6.3.4. Rectocele

**Figure 11:**
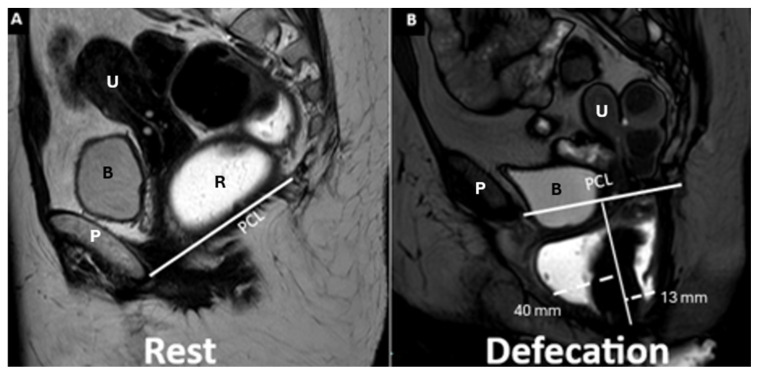
Sagittal midline image of the anterior/posterior rectocele during evacuation, illustrating both anterior and posterior rectoceles. The anorectal wall appears normal at rest (**A**), but during evacuation (**B**) the white dotted lines highlight the extent of the rectocele—40 mm anteriorly and 13 mm posteriorly. The PCL (white continuous), and anterior/posterior rectocele (white dotted) are depicted. Identifiable structures include the pubic symphysis (P), rectum (R), bladder (B), and uterus (U).

Rectocele is characterized by an abnormal bulging of the rectal wall on straining relative to the resting state. Rectoceles can be classified as anterior, posterior, or lateral (Figure 11) [70]. Anterior rectocele is the most common [83]. Less frequent posterior rectocele is expressed as an outpouching of the posterior rectal wall through a defect in the levator ani, often with disruption of the anococcygeal ligament [84]. Lateral rectoceles, also resulting from disruption of the rectovaginal fascia, may not be evident on midline sagittal images [13], highlighting the value of additional coronal dynamic imaging within MRD protocols.

The presentation clinically ranges from perineal and vaginal pressure to constipation and inability to completely empty the bowel, which requires transvaginal manual reduction [57]. The Pelvic Organ Prolapse Quantification System [72] is being increasingly used for the quantification of pelvic floor defects. Physical examination, however, can provide incorrect diagnoses, particularly in obese, poorly mobile, or uncooperative patients [31]. Additionally, rectocele may be accompanied by cul-de-sac hernias such as enterocele, peritoneocele, or sigmoidocele that cannot be distinguished on clinical examination.

MRD can reveal rectocele on defecation or on Valsalva maneuvers, and sagittal T2-weighted images can assess its size on the anteroposterior plane [16,84]. Consensus opinion recommends against using outdated grading systems, as small rectoceles (<2 cm) are often found in asymptomatic women and may instead reflect posterior vaginal wall displacement and rectal emptying following evacuation [85].

Management depends on symptoms of severity. Initial treatment includes dietary modifications (fiber and fluid intake) and biofeedback for dyssynergia. Surgery is reserved for refractory cases [85].

#### 6.3.5. Anterior Rectocele

**Figure 12:**
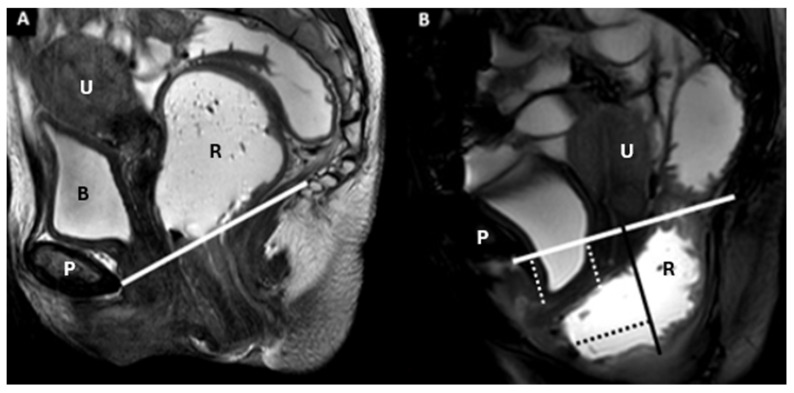
MRI at rest (**A**) and during defecation (**B**) showing isthmocele, cystocele, and anterior rectocele. The M line (dark continuous) and PCL (white continuous) are marked. The cystocele and anterior isthmocele are indicated by white dotted lines, while the anterior rectocele is highlighted with black dotted lines. Identifiable structures include the pubic symphysis (P), rectum (R), bladder (B), and uterus (U).

Anterior rectocele is the most common posterior compartment disorder and causes extrinsic mass effect of the rectum on the posterior vaginal wall by either stretching or rupturing of the rectovaginal fascia, often accentuated after gynecological interventions (Figure 12) [70]. It presents as a bulge of the anterior rectal wall into the posterior vaginal wall, leading to obstructed defecation, manual evacuation maneuvers, and pelvic pressure [83, 84]. MRD is crucial for differentiating clinically significant rectoceles (which are classified on sagittal images as mild if <2 cm, moderate if 2–4 cm, and severe if >4 cm; see Table 5) from asymptomatic incidental findings, which may occur in up to 80% of women [86].

#### 6.3.6. Rectal Intussusception

Rectal intussusception or rectal invagination, with or without external prolapse, is frequently associated with pelvic floor disorders after pelvic surgery. Intussusception can cause obstruction of rectal contents and may remain internal (intra-rectal), extend into the anal canal (intra-anal), or pass beyond the sphincter (extra-anal or extra-rectal), often referred to as rectal prolapse [87].

Symptoms of rectal intussusception include obstructive defecation, fecal incontinence, or rectal bleeding, which initially occur only during defecation but can eventually take place with minimal straining or even in the upright position with advancing pelvic laxity [88]. MRD, which typically shows intussusception at the end of evacuation, is important for diagnosis and grading (intra-rectal, intra-anal, or extra-anal) on the Oxford system (Table 6) to guide treatment planning, especially in those with impaired evacuation or if intussusception is into the anal canal apex [88].

#### 6.3.7. Dyssynergia

Dyssynergia is characterized by delayed onset of defecation or expulsion of less than one-third of rectal contents in 60 s, characteristically with reduced ano-rectal angle, paradoxical contraction of the anal sphincter, or minimal pelvic floor descent on Valsalva maneuver and defecation [89]. Dyssynergia leads to pain in the rectum, straining for a prolonged period, and inadequate evacuation, and is a functional defecation disorder with paradoxical contraction or failure to relax the puborectalis muscle or the anal sphincter [84,85]. MRD is especially valuable in postoperative patients and uncertain or severe cases as it images both structural and functional derangements—such as cul-de-sac hernias and rectal intussusception—with classic features such as paradoxical puborectalis contraction, anorectal angle narrowing on attempts at defecation, anterior abdominal wall bulging, and M-line elongation leading to evacuation difficulty [88].

## 7. Current Challenges of MR Defecography

MRD possesses many advantages but many disadvantages that could affect universal implementation into clinical practice. One major limitation is that there are no standard reporting guidelines, which leads to inconsistency in interpretation and reduces the generalizability of results across institutions [56]. Such inconsistency can hinder interprofessional teamwork and communication and conceal surgical planning. To resolve this problem, supplementing template structured reports with objective prolapse measurements, pragmatic parameters, and standardized postoperative vocabulary is strongly recommended to enhance diagnostic consistency and accuracy [62]. The high cost and limited access to MRD, especially in resource-poor environments, and a narrow evidence base for both prognostic and therapeutic use help to highlight the imperative need for cost-effectiveness research, protocol optimization, and large-scale multicenter studies to determine its use in guiding management and in predicting surgical outcomes in different patient populations [63]. Finally, MRD is unfeasible in patients with certain implants, severe claustrophobia, or limited mobility—hence, fluoroscopic defecography must be preferred in these cases—and collaboration between radiologists, gynecologists, colorectal surgeons, and data analysts is crucial to refine predictive models and implement them into clinical practice [5].

## 8. Needs for Future Research

Future research will need to focus on the integration of more recent imaging sequences and technology, such as diffusion-weighted imaging (DWI) and functional MRI (fMRI), into attempts at improving postoperative assessment. DWI can potentially be beneficial for the assessment of tissue microstructure in that it shows alterations in the movement of water molecules, which can prove beneficial for detecting postoperative fibrosis, inflammation, or residual disease not identified using routine sequences [90]. fMRI, on the other hand, can assist in assessing the functional integrity of pelvic floor muscles and the corresponding neural pathways through real-time brain–pelvic floor connectivity patterns during straining or contraction, thus providing insight into both structural and neurological recovery following surgery [91]. Additional sequences would allow improved discrimination among soft tissues as well as, perhaps, even improved diagnosis. Coronal images, although useful for anatomic orientation and for detection of lateral compartment defects, are functionally limited for pelvic floor evaluation. MRI sagittal images in defecography are superior for the quantitation of disturbances because they show dynamic visualization of organ descent, pelvic floor movement, and anorectal angle alteration during straining, therefore providing more accurate functional evaluation. In addition, the use of artificial intelligence in imaging interpretation and risk stratification would enable the standardization of measurements and reporting. Further cost-effectiveness studies need to evaluate the impact of MRD on outcomes and its worth in the longer term for patient care improvement. Multicenter trials must be conducted rigorously in order to validate predictive models in diverse patients so that applicability and generalizability are ensured.

## 9. Conclusions

MR defecography represents a crucial advancement in the imaging of pelvic floor disorders in the gynecologic postoperative patient. With the ability for dynamic, multi-compartmental imaging, exquisite soft tissue contrast, and accurate assessments of surgical changes, it is an invaluable tool for both preoperative assessment and postoperative surveillance. While challenges such as reporting heterogeneity and accessibility remain, continued research and standardization will further intensify its role in optimizing patient outcomes. By integrating MR defecography into a clinical pathway and fostering interdisciplinary communication, clinicians can enhance the diagnostic precision of pelvic floor dysfunction and tailor interventions to the individualized requirements of each patient, ultimately optimizing the quality of postoperative care in gynecologic surgery.

## Figures and Tables

**Table 1:** Summary of the endopelvic fascia, pelvic diaphragm, and urogenital diaphragm, highlighting key muscles like the levator ani and their roles in maintaining pelvic organ support.

Layer	Description	Key Muscles or Structures
Endopelvic fascia	Superior layer enveloping the pelvic diaphragm	Supports pelvic organs
Pelvic diaphragm	Composed of ischiococcygeus, iliococcygeus, pubococcygeus, and puborectalis	Forms the levator ani, maintains pelvic support
Urogenital diaphragm	Triangular layer, anterior and middle compartments	Encloses the urethral sphincter and the perineal space

**Table 2:** Key imaging modalities for pelvic floor dysfunction, highlighting primary indications, limitations, specific POP defects diagnosed, and how each guides treatment planning. It supports tailored diagnostic and management strategies in urogynecological practice.

Imaging Technique	Indications	Limitations	POP Defects	Treatment Guidance
Urodynamics	Bladder function, voiding	Invasive, limited anatomy	Stress urinary incontinence	Conservative vs. surgical
Video-UDS/Fluoroscopy	Incontinence, hypermobility, prolapse	Radiation, anatomy limits	Cystocele, rectocele, hypermobility	Surgical planning
Cystoscopy	Injury, intraoperative check	Invasive, limited scope	Fistulas, erosion	Intraoperative safety, repair guidance
Ultrasound	Real-time imaging, mobility	Operator-dependent, limited depth	Anterior/Posterior/Apical defects	Conservative or surgery referral
3D Ultrasound	Levator, implants, mesh	Cost, training, access	Levator avulsion, mesh issues	Surgical planning refinement
Dynamic MRI	Multicompartment, planning	Cost, availability	Vault prolapse, complex defects	Advanced surgical planning

**Table 3:** MR pelvic floor imaging protocol, detailing imaging planes, sequences, phases, and field of view for comprehensive functional assessment.

Imaging Plane	Sequence	Phase	Field of View
Axial	T2 smFOV	Resting	Rectum through the gluteal folds
Coronal	T2 smFOV	Resting	Sacrum through pubic symphysis
Sagittal	T2 oblique	Resting	Slices through anal canal; rectum distended with ultrasound gel.
Sagittal	T2 oblique	Kegel	Slices through anal canal
Sagittal	FIESTA or SSFSE dynamic	Defecation	Coccyx through pubic symphysis; start scan and after 5 s, ask the patient to defecate. Ensure the patient fully empties the rectum. Repeat if necessary to capture complete evacuation dynamics.
Coronal	T2 breath hold	Valsalva	Sacrum to pubic symphysis

**Table 4:** Key anatomical reference lines and staging criteria for pelvic organ prolapse (POP) used in MRI evaluation of pelvic floor dysfunction. This table outlines the definitions, clinical roles, and severity classifications for the pubococcygeal line (PCL), mid-pubic line (MPL), H Line (hiatal line), M Line (muscle line), and anorectal angle. These parameters provide a standardized approach to assessing puborectal hiatus, posterior organ descent, and prolapse severity, facilitating accurate diagnosis and classification of pelvic floor disorders. Values and staging criteria were provided according to García del Salto et al. [57].

Component/Reference Line	Definition	Role in HMO System	Normal Range/Severity/Staging
Pubococcygeal Line (PCL)	Line from the inferior pubic border to the last coccygeal joint	Baseline for measuring organ descent	PCL Compartment Staging Stage 0: Above PCL Stage I: Descent <3 cm below PCL Stage II: Descent 3–6 cm below PCL Stage III: Descent >6 cm below PCL Stage IV: Complete organ prolapse
Mid-pubic Line (MPL)	Line drawn through and caudad through the axis of the mid-pubic symphysis on sagittal MRI	Used to assess pelvic organ prolapse (POP); a 90° angle is measured between MPL and the bladder, vaginal vault, and anterior anorectal junction	MPL Compartment Staging Stage 0: >3 cm above MPL or TVL −2 cm Stage I: 1 cm above ≤ X ≤ 1 cm below MPL Stage II: 1 cm above ≤ X ≤ 1 cm below MPL Stage III: ≥1 cm below MPL Stage IV: Complete organ prolapse
H Line (Hiatal Line)	Distance between the inferior pubic border and the anorectal junction	Assesses puborectal hiatus (anteroposterior dimension during straining)	POP Grade Hiatal Enlargement Normal: <6 cm Mild: 6–8 cm Moderate: 8–10 cm Severe: >10 cm
M Line (Muscle Line)	Perpendicular line from the PCL, measuring organ descent	Evaluates posterior pelvic organ descent	Pelvic Floor Descent Normal: <2 cm Mild: 2–4 cm Moderate: 4–6 cm Severe: >6 cm
Anorectal Angle	Angle between the posterior distal rectum and the anal canal’s central axis	Reflects the levator ani muscle function during contraction	108–127° at rest, decreases by 15–20° during contraction

**Table 5:** This table summarizes the pelvic compartments and their contained organs, supportive structures, and associated disorders. It also outlines MRI measurement methods used to assess pelvic organ prolapse, incorporating grading criteria for organ descent based on the pubococcygeal line (PCL). Values and classifications are reported according to García del Salto et al. [56].

Pelvic Compartment	Contained Organs	Supportive Structures	Condition	Measurement Method	Grading Criteria
Anterior	Urinary bladder, urethra	Pubocervical fascia (part of endopelvic fascia)	Cystocele (bladder descent)	Bladder neck position relative to PCL	Mild: 1–3 cm, Moderate: 3–6 cm Severe: >6 cm
Middle	Vagina, cervix, uterus	Paracolpium, parametrium (endopelvic fascia)	Uterine descent	Uterine fundus position below PCL	Mild: 1–3 cm Moderate: 3–6 cm Severe: >6 cm
			Vaginal descent	Vaginal fornix position relative to PCL	Mild: 1–3 cm Moderate: 3–6 cm Severe: >6 cm
Posterior	Colon		Enterocele, sigmoidocele, peritoneocele	Position below the posterior cervicovaginal ligament	Mild: 1–3 cm Moderate: 3–6 cm Severe: >6 cm
	Rectum, anal canal	Rectovaginal fascia, perineal body	Rectal intussusception/prolapse	Extent of rectal mucosa relative to rectocele/anal canal	Mild: 1–2 cm Moderate: 2–4 cm Severe: >4 cm

**Table 6:** Oxford Internal Rectal Prolapse Grading System—The Oxford grading system classifies rectal prolapse based on the degree of mucosal or full-thickness prolapse and its impact on defecation.

Type	Grade	Description
Internal Prolapse (Mucosal or Full-Thickness Intussusception)	Low-Grade (Grade 1–2)	Mucosal folds are visible or prolapse into the rectal lumen but do not obstruct defecation.
	High-Grade (Grade 3–4)	Mucosal or full-thickness prolapse causes partial or significant obstruction, leading to defecatory dysfunction.
External Prolapse	Grade 5	Full-thickness rectal prolapse extends beyond the anal verge, visible externally.

## Data Availability

No new data were created or analyzed in this study.
